# Quantitative assessment of PSMA PET response to therapy in castration-sensitive prostate cancer using an automated imaging platform for disease identification and measurement

**DOI:** 10.1186/s41824-023-00165-6

**Published:** 2023-04-03

**Authors:** Sai Duriseti, Gholam Berenji, Sonny Tsai, Matthew Rettig, Nicholas G. Nickols

**Affiliations:** 1 VA Greater Los Angeles, Radiation Oncology Service, 11301 Wilshire Blvd, Building 500, Suite 0426, Los Angeles, CA 90073 USA; 2 Departments of Radiation Oncology, University of California, Los Angeles, 200 UCLA Medical Plaza, Suite B265, Los Angeles, CA 90095 USA; 3 VA Greater Los Angeles, Nuclear Medicine Service, 11301 Wilshire Blvd, Building 500, Suite 0090, Los Angeles, CA 90073 USA; 4 Radiological Sciences, University of California, Los Angeles, 200 Medical Plaza, Suite B-114, Los Angeles, CA 90095 USA; 5 Greater Los Angeles VA, Hematology and Oncology Section, 11301 Wilshire Blvd. Building 304, Suite E2-218, Los Angeles, CA 90073 USA; 6 Department of Urology, University of California, Los Angeles, 200 Medical Plaza, Suite 140, Los Angeles, CA 90095 USA; 7 Department of Medicine, University of California, Los Angeles, 200 Medical Plaza, Suite 140, Los Angeles, CA 90095 USA

**Keywords:** PSMA, Automated, Artificial intelligence

## Abstract

**Rationale:**

Prostate cancer treatment response may be automatically quantified using a molecular imaging analysis platform targeting prostate-specific membrane antigen (PSMA).

**Methods:**

A retrospective analysis of patients with castration-sensitive prostate cancer who underwent PSMA-targeted molecular imaging prior to and 3 months or more after treatment was conducted. Disease burden was analyzed with aPROMISE, an artificial intelligence imaging platform that automatically quantifies PSMA-positive lesions. The calculated PSMA scores for prostate/bed, nodal, and osseous disease sites were compared with prostate-specific antigen (PSA) values.

**Results:**

Of 30 eligible patients, the median decline in prostate/bed, nodal, and osseous disease PSMA scores were 100% (range 52–100%), 100% (range − 87–100%), and 100% (range − 21–100%), respectively. PSMA score decline was significantly associated with PSA decline.

**Conclusion:**

Changes in aPROMISE PSMA scores are associated with changes in PSA and may quantify treatment response.

## Introduction

High-risk localized, recurrent, or metastatic CSPC patients receive androgen receptor (AR) axis inhibition and local therapy to disease sites. PSA changes traditionally gauge treatment response, but cannot discern lesion-level response (Scher et al. 2016). Currently employed imaging biomarkers allow noninvasive disease measurement using standard imaging modalities, but suffer limitations despite anatomy-specific quantitative techniques (Schwartz et al. 1990; van Persijn van Meerten 2010; Ulmert et al. 2012; Mitsui et al. 2012; Anand et al. 2016; Anand et al. 2020).

Disease-specific molecular imaging with PSMA-targeted radiotracers allows quantitative spatial resolution of individual tumors with positron emission tomography (PET) when combined with computed tomography (CT). PSMA PET-CTs are increasingly used for prostate cancer staging and can quantify treatment response (Fanti et al. 2020; Gafita et al. 2022). [^18^F]DCFPyL is a PSMA-targeted radiotracer with exceptional sensitivity and specificity for lesion detection (Morris et al. 2021; Pienta et al. 2021). Prostate cancer molecular imaging standardized evaluation (PROMISE) criteria provides a standardized framework for classifying and quantifying PSMA tracer-avid disease (Eiber et al. 2018). Automated PROMISE (aPROMISE) is an AI platform that enhances PROMISE’s approach by auto-segmenting organs, quantifying radiotracer uptake in reference organs, identifying pathologic lesions, and quantifying lesion uptake to facilitate calculation of PSMA scores that take into account both lesion volume and standardized uptake values (SUV) in specific anatomic regions (Nickols et al. 2022; Johnsson et al. 2022).

## Materials and methods

Clinical data for CSPC patients treated from 2018 onwards were reviewed. Patients who had both baseline and follow-up PSMA PET-CTs at least 3 months after treatment initiation were identified. PSMA PET-CTs were read by a nuclear medicine physician for baseline and follow-up disease assessment.

**Fig. 1:**
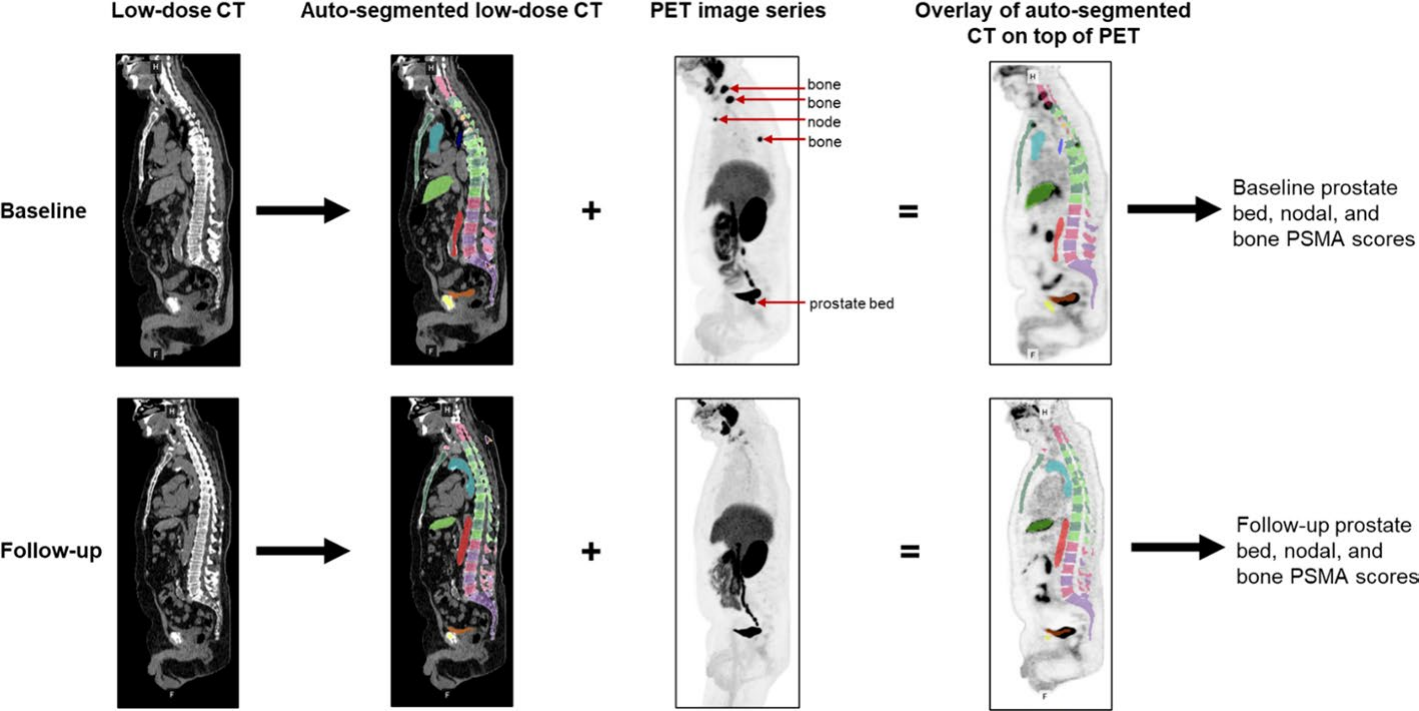
aPROMISE workflow. Anatomy is auto-segmented, radiotracer avid lesions are identified and auto-segmented, and then, the CT and PET image sets are overlaid to obtain annotated and quantifiable lesions. Sagittal PSMA PET-CT slices before and after therapy for a patient are shown

**Fig. 2:**
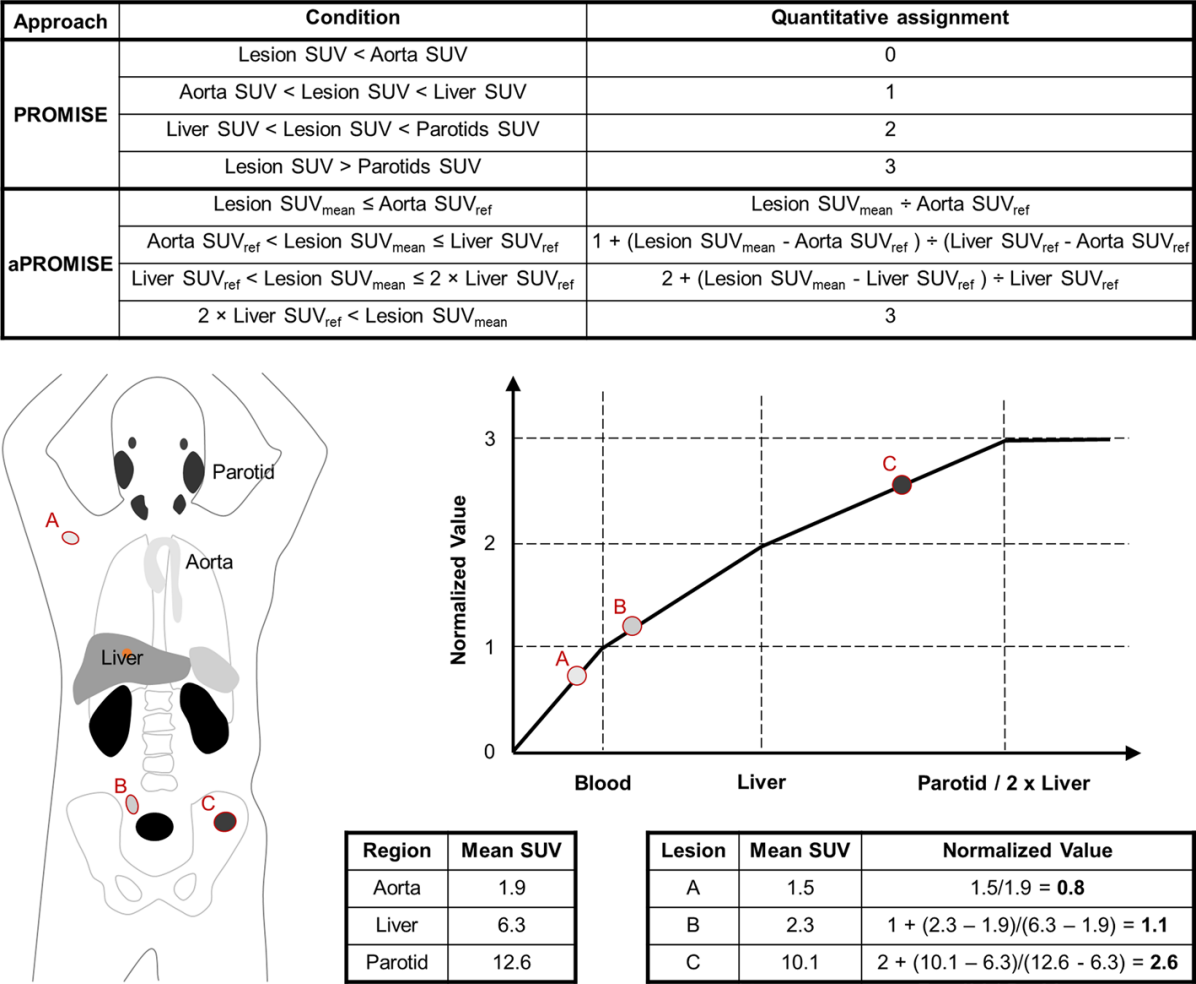
PROMISE and aPROMISE uptake value normalization comparison with schematic example of aPROMISE normalized SUV calculation

aPROMISE was used to identify, quantify, and calculate changes in PSMA tracer-avid disease (Fig. 1) (Calais et al. 2022). Patient anatomy was auto-segmented from the low-dose CT portion of the PSMA PET-CT using a convolutional neural network (CNN) trained on CTs with expertly defined anatomy. Auto-segmented liver and aorta structures were overlayed on the PET dataset to calculate reference standard uptake values (SUVs_ref_) from the liver and aorta SUVs_mean_, which were then used to identify other normal tissues. Putative lesion identification was divided into hot spot detection and segmentation phases. Hot spot detection utilizes a second CNN trained on expertly annotated PSMA PETs. The segmentation phase uses an adaptive threshold based on contextual information from the two deployed CNNs (Fig. 2). The hot spot detection algorithm has high sensitivity to minimize the number of lesions that miss detection and require manual contouring. A nuclear medicine physician assists the process by reviewing auto-segmented lesions to identify true versus fals positives. A PSMA score for the selected set of lesions was calculated as the product of the individual lesion volume and SUV_mean_, normalized by the SUV_mean_ of reference tissue. An anatomic compartment PSMA score for prostate/bed, nodal, or osseous disease was calculated as the sum of individual lesion PSMA scores in the compartment (e.g.,$\mathop {\Sigma }\limits_{{{\text{Lymph}}\;{\text{Nodes}}}} {\text{PSMA}}\;{\text{score}} \times {\text{Uptake}}\;{\text{Volume}} = {\text{Lymph}}\;{\text{Node}}\;{\text{PSMA}}\;{\text{Score}}$). An additional composite score was calculated as a sum of the compartment PSMA scores. A Pearson’s R test was performed for total PSMA scores, as well as for the prostate/bed, nodal, and osseous compartment PSMA scores. Significance was determined by a 2-tailed T test.

## Results

Thirty patients were eligible for analysis. All patients demonstrated a decline in PSA before the second PSMA PET-CT. The median interval between PSMA PET-CTs was 8 months (range: 3–30). Baseline prostate/bed, regional nodal, non-regional nodal, and osseous disease was present in 27, 25, 9, and 18 patients, respectively. Treatment details were available for 29 patients. Of these, 4 patients had prior prostatectomy with 1 patient experiencing bed recurrence. Patient clinicopathologic data, treatment course, and biochemical outcome information are shown in Table 1.

**Table 1:** Patient clinicopathologic data and treatment course

Patient number	Disease timing	miTNM at diagnosis	Prostate/bed disease	Regional nodal disease	Non-regional Nodal diseaseˤ	Osseous disease	Imaging interval (months)	Interval treatment	% reduction in PSA
1	De novo	T3bN1bM0	Yes	Yes	No	No	30	ADT + radiotherapy	100
2	De novo	T2mN0M1b	Yes	No	No	Yes	14	Surgery + ADT + radiotherapy	100
3	De novo	T2uN0M0	Yes	No	No	No	13	Surgery	84
4	Recurrence	T2mN1bM0	Yes	Yes	No	No	10	ADT + radiotherapy	99
5	De novo	T2mN1bM1b	Yes	Yes	No	Yes	21	ADT + radiotherapy	100
6	Recurrence	T2mN0M1b	Yes	No	No	Yes	3	ADT + radiotherapy	100
7	De novo	T2mN1aM0	Yes	Yes	No	No	3	Surgery	92
8	De novo	T3bN1aM1a	Yes	Yes	Yes	No	20	Surgery + ADT + radiotherapy	95
9	De novo	T3bN1bM1a	Yes	Yes	Yes	No	21	Surgery + ADT	92
10	De novo	T3aN1aM1b	Yes	Yes	No	Yes	11	ADT + radiotherapy	100
11˘	Recurrence	T0N0M1b	No	No	Yes	Yes	12	ADT + radiotherapy	100
12^˜^	Recurrence	T0N1aM0	No	Yes	No	No	12	Unknown˜	Unknown˜
13	De novo	T3bN1bM1b	Yes	Yes	Yes	Yes	4	ADT	69
14 ^ˠ^	De novo	T3bN1bM1b	Yes	Yes	No	Yes	5	ADT + radiotherapy	100
15	De novo	T3bN0M1b	Yes	Yes	Yes	Yes	7	ADT + radiotherapy	83
16	De novo	T3bN1bM1b	Yes	Yes	No	Yes	7	ADT + radiotherapy	98
17˘	Recurrence	TrN1bM1b	Yes	Yes	No	Yes	15	ADT + radiotherapy	100
18	De novo	T2uN1aM1b	Yes	Yes	No	Yes	26	Surgery + ADT + radiotherapy	100
19	De novo	T3bN1bM1b	Yes	Yes	No	Yes	7	ADT + radiotherapy	100
20	De novo	T2mN1aM1b	Yes	Yes	No	Yes	7	ADT + radiotherapy	100
21	De novo	T3bN1aM1b	Yes	Yes	No	Yes	7	ADT + radiotherapy	100
22	De novo	T2mN1aM0	Yes	Yes	No	No	7	ADT + radiotherapy	100
23	De novo	T2mN1bM1a	Yes	Yes	Yes	No	7	ADT + radiotherapy	100
24	De novo	T3bN1bM1b	Yes	Yes	No	Yes	18	ADT + radiotherapy	100
25	De novo	T2mN0M1b	Yes	No	No	Yes	9	ADT + radiotherapy	100
26	De novo	T2mN1aM0	Yes	Yes	No	Yes	6	ADT + radiotherapy	99
27	De novo	T2mN0M1b	Yes	No	No	Yes	7	ADT + radiotherapy	100
28˘	Recurrence	T0N1bM1a	No	Yes	Yes	No	7	ADT	100
29	De novo	T2mN1bM1a	Yes	Yes	Yes	No	8	ADT + radiotherapy	100
30	De novo	T2mN1bM1a	Yes	Yes	Yes	No	7	ADT + radiotherapy	97

**˘**Patients with recurrent disease who had undergone prior prostatectomy

˜Patient 12’s treatment course and PSA response was not available for review

**ˠ**Patient 14 had concurrent lung metastases at initial PSMA PET-CT

ˤNon-regional nodal disease was any nodal disease above the bifurcation of the common iliac arteries

Changes in composite and compartment-specific PSMA scores are shown in Table 2. Median prostate/bed, nodal, osseous, and composite PSMA scores at baseline were 21.6 (range: 0.9–150.5), 5.3 (range: 0.1–105.6), 2.2 (0.1–96.2), and 9.7 (0.2–106.8), respectively. Median PSMA scores for prostate/bed, nodal, osseous, and composite at follow-up PSMA PET-CT were 0 (0–6.2), 0 (0–91.1), 0 (0–36.2), and 0 (0–91.6), respectively. Baseline prostate/bed PSMA scores were significantly correlated with baseline PSA values (*p* < 0.001); however, neither nodal (*p* = 0.53) nor osseous (*p* = 0.65) baseline PSMA scores were correlated with baseline PSA values.

**Table 2:** The percent decrease in each PSMA score was calculated from the quantified pre-treatment and post-treatment PSMA PET-CT for the total composite and each anatomic compartment

Patient number	Lymph node % change	Osseous disease % change	Primary (prostate or prostate bed) % change	Total composite % change	% decrease PSA
1	− 100%	N/A	− 100%	− 100%	− 100
2	N/A	14%	− 100%	− 97%	− 100
3	N/A	N/A	− 100%	− 100%	− 84
4	− 66%	N/A	− 100%	− 70%	− 99
5	− 100%	− 47%	− 100%	− 100%	− 100
6	N/A	− 87%	− 100%	− 89%	− 100
7	N/A	N/A	− 100%	− 100%	− 92
8	− 100%	N/A	− 100%	− 100%	− 95
9	− 62%	N/A	− 100%	− 94%	− 92
10	− 100%	− 100%	− 100%	− 100%	− 100
11	− 100%	− 75%	N/A	− 76%	− 100
12˜	− 20%	N/A	N/A	− 20%	Unknown
13	87%	− 45%	− 100%	31%	− 69
14ˠ	− 96%	− 62%	− 100%	− 70%	− 100
15	N/A	− 35%	− 100%	− 98%	− 83
16	− 100%	− 80%	− 41%	− 49%	− 98
17	− 100%	− 100%	N/A	− 100%	− 100
18	N/A	− 100%	− 100%	− 100%	− 100
19	− 100%	− 100%	− 100%	− 100%	− 100
20	N/A	N/A	− 100%	− 100%	− 100
21	− 100%	− 64%	− 100%	− 98%	− 100
22	− 100%	− 100%	− 100%	− 100%	− 100
23	− 100%	− 100%	− 100%	− 100%	− 100
24	− 100%	− 100%	− 100%	− 100%	− 100
25	N/A	− 100%	− 100%	− 100%	− 100
26	− 100%	− 100%	− 95%	− 98%	− 99
27	N/A	− 100%	− 95%	− 95%	− 100
28	− 59%	N/A	N/A	− 59%	− 100
29	− 100%	N/A	− 100%	− 100%	− 100
30	− 100%	N/A	− 52%	− 56%	− 97
Pearson R	0.95	0.38	− 0.06	0.61	
*p* value	4 E-10	0.09	Not calculated	5 E-4	

A Pearson R correlation coefficient was calculated between the percent decrease in the PSMA score, and PSA was calculated and is shown. “N/A” indicates that the patient did not have relevant disease that could be scored for that anatomic compartment

˜Patient 12’s treatment course and PSA response was not available for review

**ˠ**Patient 14 had concurrent lung metastases at initial PSMA PET-CT

**Fig. 3:**
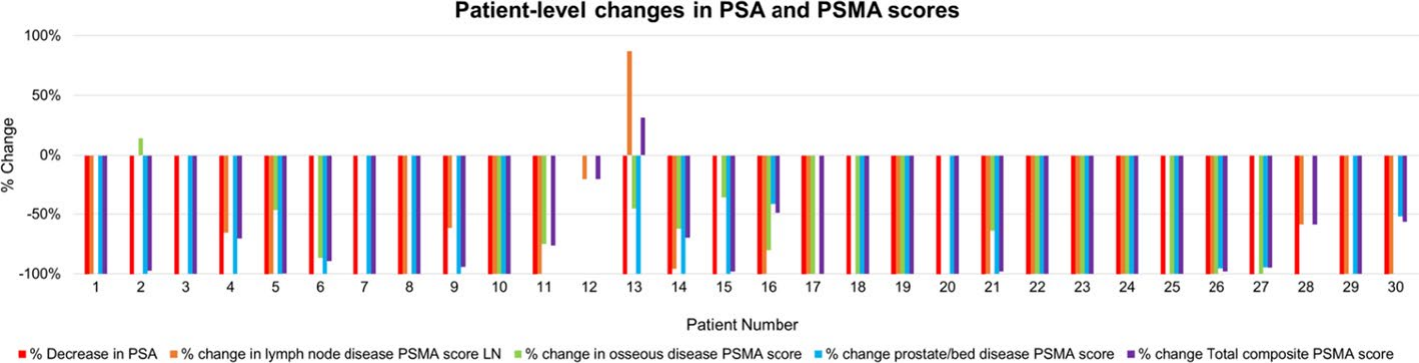
Percent change in PSA and prostate/bed, nodal, and osseous disease PSMA scores for each patient

**Fig. 4:**
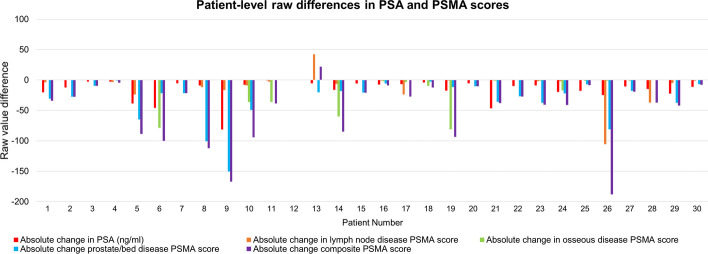
Absolute raw changes in PSMA scores and PSA after treatment

The median PSA decrease was 100% (range: 68–100%). Changes in PSMA scores were significantly correlated with corresponding decreases in PSA for composite and nodal disease, but not for prostate/bed or osseous disease (Table 2). Patient-level changes in PSMA scores and PSA are shown in Fig. 3. Patient 2 and Patient 13 had an increase in osseous, and nodal and composite PSMA scores, respectively, despite PSA decline after treatment. The osseous and prostate PSMA scores before/after treatment for Patient 2 were 0.62/0.70 and 27.5/0, respectively; thus, the before/after composite PSMA score of 28.1/0.7 was only minimally affected by increased osseous disease uptake. The osseous, lymph node, and prostate PSMA scores before/after treatment for Patient 13 were 0.99/0.48, 48.71/91.06, and 20.12/0, respectively; thus, the before/after composite PSMA score of 69.72/91.55 was heavily weighted by increased nodal disease uptake. Absolute changes in PSMA scores are shown in Fig. 4.

## Conclusion

Changes in prostate cancer disease burden as identified by PSMA-targeting radiotracers correlate with PSA response, but few studies have shown correlation between PSA changes and qualitative PET-based disease response (Hope et al. 2017; Schmidkonz et al. 2018; Zacho and Petersen 2018; Afshar-Oromieh et al. 2018; Aggarwal et al. 2018; Ettala et al. 2020; Emmett et al. 2019; Shagera et al. 2022). For instance, RECIP 1.0 assesses response for patients with castration resistant prostate cancer treated with targeted radionuclide therapy (Gafita et al. 2022). Here we present a method to automatically annotate and measure lesion changes for patients with CSPC using a previously validated tool for PSMA radiotracer uptake quantification. While there are no prospective data to demonstrate utility of PSMA-targeted PET-CTs for response assessment, the quantifiable PSMA score changes in this study were significantly correlated with PSA decline (Fanti et al. 2021).

PSMA expression is down-regulated by AR axis activation and up-regulated by its suppression, which may complicate response assessment of immediate post-treatment PSMA PET-CTs (Hope et al. 2017; Lückerath et al. 2018). Initial responses to AR-directed therapy as assessed on PSMA PET-CT were previously evaluated in small studies with variable time courses (Hope et al. 2017; Zacho and Petersen 2018; Aggarwal et al. 2018; Ettala et al. 2020; Emmett et al. 2019; Wondergem et al. 2020; Plouznikoff et al. 2019). These studies report inter-patient, intra-patient, and intra-patient inter-lesion radiotracer uptake heterogeneity after AR-directed therapy, which further complicates global interpretation of treatment efficacy. Discordance between changes in anatomically stratified PSMA scores and PSA were also observed here. Patients 2 and 13 had PSMA score increases. While the percent change in osseous PSMA score for Patient 2 was + 14%, this represented a raw change from 0.6 to 0.7. By contrast, Patient 13 had an increase of the nodal PSMA score of 87%, which represented a raw change from 48.7 to 91.1. This impacted the composite PSMA score and was qualitatively appreciated on imaging. Thus, utilization of PSMA score percent change may only be relevant above a certain raw score threshold. These cases also demonstrate how PSA changes may not reflect disease response at the individual lesion level, thus underscoring the need for imaging biomarkers that assess response at the lesion level.

It remains unclear whether aPROMISE may serve as an imaging biomarker and how these data affect clinical outcomes or provider decision-making. Additionally, as our approach was geared toward high sensitivity for lesion detection, clinical guidance to determine true versus false positive disease is required. The putative concordance between PSA and PSMA imaging response justifies PSMA PET-CT inclusion into prospective studies as a method to stratify therapeutic approaches. Given the few discordances between PSA and PSMA score changes here, additional work to track and compare changes at the lesion-specific level to assess intra-patient, inter-lesion heterogeneity is warranted.

## Data Availability

The datasets generated and/or analyzed during the current study are not publicly available due to their nature as protected PHI of VA patients, but are available from the corresponding author on reasonable request.

## Abbreviations

PSMA: Prostate-specific membrane antigen
CSPC: Castration-sensitive prostate cancer
PSA: Prostate-specific antigen
PROMISE: Prostate cancer molecular imaging standardized evaluation
AI: Artificial intelligence
PET: Positron emission tomography
CT: Computed tomography
SUV: Standardized uptake value
CNN: Convolutional neural network
